# Complex Spatiotemporal Responses of Global Terrestrial Primary Production to Climate Change and Increasing Atmospheric CO_2_ in the 21^st^ Century

**DOI:** 10.1371/journal.pone.0112810

**Published:** 2014-11-17

**Authors:** Shufen Pan, Hanqin Tian, Shree R. S. Dangal, Chi Zhang, Jia Yang, Bo Tao, Zhiyun Ouyang, Xiaoke Wang, Chaoqun Lu, Wei Ren, Kamaljit Banger, Qichun Yang, Bowen Zhang, Xia Li

**Affiliations:** 1 International Center for Climate and Global Change Research, School of Forestry and Wildlife Sciences, Auburn University, Auburn, Alabama, United States of America; 2 State Key Laboratory of Urban and Regional Ecology, Research Center for Eco-Environmental Sciences, Chinese Academy of Sciences, Beijing, China; 3 State Key Laboratory of Desert and Oasis Ecology, Xinjian Institute of Ecology and Geography, Chinese Academy of Sciences, Urumqi, China; Montana State University, United States of America

## Abstract

Quantitative information on the response of global terrestrial net primary production (NPP) to climate change and increasing atmospheric CO_2_ is essential for climate change adaptation and mitigation in the 21^st^ century. Using a process-based ecosystem model (the Dynamic Land Ecosystem Model, DLEM), we quantified the magnitude and spatiotemporal variations of contemporary (2000s) global NPP, and projected its potential responses to climate and CO_2_ changes in the 21^st^ century under the Special Report on Emission Scenarios (SRES) A2 and B1 of Intergovernmental Panel on Climate Change (IPCC). We estimated a global terrestrial NPP of 54.6 (52.8–56.4) PgC yr^−1^ as a result of multiple factors during 2000–2009. Climate change would either reduce global NPP (4.6%) under the A2 scenario or slightly enhance NPP (2.2%) under the B1 scenario during 2010–2099. In response to climate change, global NPP would first increase until surface air temperature increases by 1.5°C (until the 2030s) and then level-off or decline after it increases by more than 1.5°C (after the 2030s). This result supports the Copenhagen Accord Acknowledgement, which states that staying below 2°C may not be sufficient and the need to potentially aim for staying below 1.5°C. The CO_2_ fertilization effect would result in a 12%–13.9% increase in global NPP during the 21^st^ century. The relative CO_2_ fertilization effect, i.e. change in NPP on per CO_2_ (ppm) bases, is projected to first increase quickly then level off in the 2070s and even decline by the end of the 2080s, possibly due to CO_2_ saturation and nutrient limitation. Terrestrial NPP responses to climate change and elevated atmospheric CO_2_ largely varied among biomes, with the largest increases in the tundra and boreal needleleaf deciduous forest. Compared to the low emission scenario (B1), the high emission scenario (A2) would lead to larger spatiotemporal variations in NPP, and more dramatic and counteracting impacts from climate and increasing atmospheric CO_2_.

## Introduction

Net Primary Productivity (NPP), a balance between photosynthetic carbon (C) uptake (Gross Primary Productivity; GPP) and losses due to plant respiration, represents the net C retained by terrestrial vegetation. It is of particular importance to humans since the largest portion of the food supply comes from terrestrial NPP [1]. NPP is also an important indicator of ecosystem health and services [2], [3], and is an essential component of the global C cycle [4]. Terrestrial NPP is sensitive to multiple environmental changes including climate and atmospheric changes [5]. The IPCC Fourth Assessment (AR4) assessment indicated that global average temperature has increased by 0.74°C since the pre-industrial times and that this trend is expected to continue through the 21^st^ century [6]. In addition, atmospheric CO_2_ concentration have increased from the pre-industrial level of 280 ppm to the 2005 level of 379 ppm [6]. Comparing the 2090s with the 2000s, under the high emission scenario (A2), global mean temperature would increase by 4.6°C, while global annual precipitation would increase by 16.8%. However, the Representative Concentration Pathways (RCP’s) scenarios used in the IPCC Fifth Assessment Report (AR5) IPCC report [7] were created in a different way and span a wider range of 21^st^ century projections. There are notable differences among the IPCC SRES scenarios and the IPCC RCP scenarios. The B1 scenario is very close to the RCP 4.5 by 2100, but there is lower emissions at the middle of 21^st^ century [8]. Similarly, the A2 scenario is between the RCP 6.0 and RCP 8.5 scenarios. Projected changes in atmospheric CO_2_ concentration showed large increases under the A2 scenario, from 379.6 ppm in the 2000s to 809 ppm in the 2090s, which is equivalent to an overall increase of 113.8%. Such dramatic changes in climate and atmospheric composition would profoundly affect the NPP of terrestrial ecosystems. It is of critical importance to quantitatively analyze the contemporary pattern of global NPP and project to what extent climate change and increasing atmospheric CO_2_ in the 21^st^ century would alter the magnitude and spatiotemporal patterns of NPP across the terrestrial ecosystems [9].

Previous studies reported that climate and increasing atmospheric CO_2_ are the primary drivers for changes in global terrestrial NPP [4], [10]–[12]. Enhanced terrestrial NPP in response to increase in temperature [13] and atmospheric CO_2_ concentration [14], [15] have been suggested across a range of terrestrial ecosystems. Over the recent three decades, climate change has been the major driver of terrestrial NPP [10], [16], [17], with further benefits from CO_2_ fertilization [4], [18]. Nemani et al. [10] reported that climate change resulted in a 6% increase in global terrestrial NPP from 1982 to1999, with the largest increase in low-latitude ecosystems. Zhao and Running et al [16] reported that in the recent decade (2000–2009), high temperature has increased water stresses and autotrophic respiration in the Southern Hemisphere resulting in a decline in global NPP by 0.55 PgC. Potter et al. [17], however, reported that rapid climate warming alleviated temperature limitations in high-latitude ecosystems which led to an increase in global terrestrial NPP by 0.14 PgC during 2000–2009. Higher temperature affect plant phenology, promoting an early growth and increasing the C assimilation in temperature-limited regions [13], [19], [20] due to the acceleration of enzymatic processes. Also, increasing CO_2_ has been found to reduce stomatal conductance [14] and increase water use efficiency [21], [22]. However, the stimulated effects of temperature on NPP may also be mitigated by increasing soil water stress and respiration rates induced by temperature rise [18], [23], [24]. In addition, increased water stress reduces nutrient uptake [25] which could potentially lead to a decline in productivity [26].While there is little doubt that climate change and increasing atmospheric CO_2_ are the primary drivers of terrestrial NPP for the recent decade, the relative contribution of different drivers in the future is still unclear.

Process-based ecosystem models are effective tools for future projection of terrestrial NPP in response to global change [27]. Various process-based ecosystem models have been developed to estimate NPP response to changes in climate and increasing atmospheric CO_2_ concentration at several scales from continental to global for both contemporary and future climatic conditions [4], [28]. Previous modeling studies found that climate change resulted in an overall decline in global NPP, but doubling atmospheric CO_2_ concentration resulted in an increase in global NPP by 16–25% [4], [18]. In a process-based model comparison study, Cramer et al. [29] found differences in global NPP among 17 models (ranging from 44.4 to 66.3 PgC yr^−1^) due largely to how the water balance was represented in models. In a similar 17-model comparison study, Friedlingstein et al. [30] found large uncertainties associated with belowground processes that resulted in different responses of NPP to global change factors across models. Thus, realistic historical assessments and future projections of global terrestrial NPP in a rapidly changing climate require more comprehensive models that include ecological, physiological and biogeochemical processes such as changes in phenology, length of growing seasons, nutrient dynamics, and ecohydrological processes.

The purpose of this study is to understand complex responses of terrestrial NPP at latitudinal, biome and global levels to projected climate change and increasing atmospheric CO_2_ in the 21^st^ century. To accomplish this task, we first established the baseline estimate of global terrestrial NPP for the first decade of the 21^st^ century by using a well-evaluated process-based ecosystem model (the Dynamic Land Ecosystem Model, DLEM [22]) driven by multiple environmental factors. Then we used the DLEM model to examine responses of terrestrial NPP to projected climate change and increasing atmospheric CO_2_ during the rest of the 21^st^ century under the IPCC Special Report on Emissions Scenarios (A2 and B1).The major objectives of this study are: (1) to estimate the contemporary global terrestrial NPP, (2) to project its changing trend in the 21^st^ century, (3) to attribute the relative contribution of climate, elevated CO_2_, and their interaction; and (4) to investigate the spatiotemporal pattern of global NPP as well as the response of different biomes to climate and CO_2_ changes.

## Methods

### 2.1 Model description

**Figure 1:**
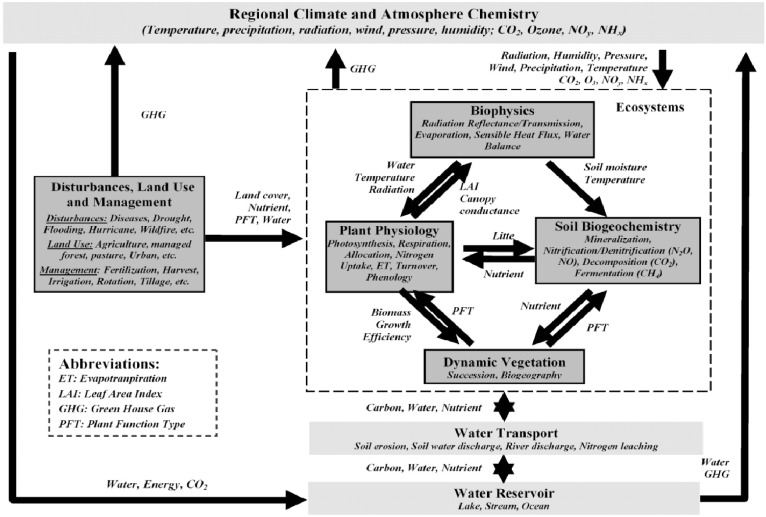
The simplified framework of Dynamic Land Ecosystem Model (DLEM) for assessing the effects of climate change and increasing atmospheric CO_2_ concentration on global terrestrial net primary production (NPP).

The DLEM is a highly integrated, process-based terrestrial ecosystem model that aims at simulating the structural and functional dynamics of land ecosystems affected by multiple factors including climate, atmospheric compositions (CO_2_, nitrogen deposition, and tropospheric ozone), land use and land cover change, and land management practices (harvest, rotation, fertilization etc). The DLEM has five core components (Figure 1): 1) biophysics, 2) plant physiology, 3) soil biogeochemistry, 4) dynamic vegetation, and 5) land use and management [22]. This model has been extensively calibrated against various field data covering forest, grassland, and cropland from the Chinese Ecological Research Network, the US Long Term Ecological Research (LTER) sites, the AmeriFlux network and other field sites. Detailed information on how DLEM simulates these processes is available in our published papers [31]–[34]. Recently, we updated the model to DLEM 2.0 version, which is characterized by cohort structure, multiple soil layer processes, coupled C, water and nitrogen cycles, multiple greenhouse (GHG) emissions simulation, enhanced land surface processes, and dynamic linkages between terrestrial and riverine ecosystems [34], [35]. Below, we briefly describe the simulation of GPP and NPP, calculation of relative CO_2_ fertilization effect, input datasets used to drive the DLEM, and global-level evaluation of simulated NPP against satellite data.

### 2.2 Modeling gross primary productivity (GPP) in the DLEM

The DLEM uses a modified Farquhar’s model to simulate GPP [36]–[39]. The canopy is divided into sunlit and shaded layers. GPP (gC m^−2^ day^−1^) is calculated by scaling leaf assimilation rates (µmol CO_2 _m^−2 ^s^−1^) up to the whole canopy:
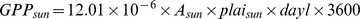


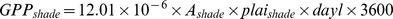


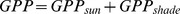



Where *GPP_sun_* and *GPP_shade_* are the GPP of sunlit and shaded canopy, respectively; A_sun_ and A_shade_ are assimilation rates of sunlit and shaded canopy; plai_sun_ and plai_shade_ are sunlit and shaded leaf area indices; dayl is daytime length (second) in a day. 12.01×10^−6^ is a constant to change the unit from µmol CO_2_ to gram C.

The *plai_sun_* and *plai_shade_* are estimated as:
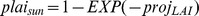


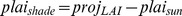



Where, *proj_LAI_* is the projected leaf area index. Using methods similar to Collatz et al. [37], DLEM determines the C assimilation rate as the minimum of three limiting rates, *w_c_*, *w_j_*, *w_e_*, which are functions that represents the assimilation rates as limited by the efficiency of photosynthetic enzymes system (Rubisco-limited), the amount of Photosynthetically Active Radiation (PAR) captured by leaf chlorophyll (light-limited), and the capacity of leaves to export or utilize photosynthesis products (export-limited) for C_3_ species, respectively. For C_4_ species, *w_e_* refer to the Phosphoenolpyruvate (PEP) carboxylase limited rate of carboxylation. The sunlit and the shaded canopy C assimilation rate can be estimated as:
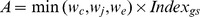


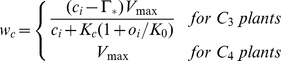


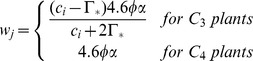


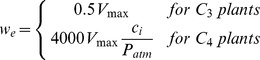
where *c_i_* is the internal leaf CO_2_ concentration (Pa); *O_i_* is the O_2_ concentration (Pa); 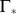 is the CO_2_ compensation point (Pa); *K_c_* and *K_o_* are the Michaelis-Menten constants for CO_2_ and O_2_, respectively; α is the quantum efficiency; *ø* is the absorbed photosynthetically active radiation (W·M^−2^); *V_max_* is the maximum rate of carboxylation varies with temperature, foliage nitrogen concentration, and soil moisture [38]:



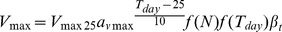
where *V_max25_* is the value at 25°C and *a_vmax_* is the temperature sensitivity parameter; *f(Tday)* is a function of temperature-related metabolic processes [36], [37].



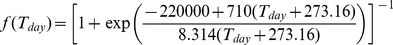

*f(N)* adjusts the rate of photosynthesis for foliage nitrogen



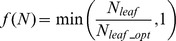



Where *N_leaf_* is the nitrogen concentration and *N_leaf_opt_* is the optimal leaf nitrogen concentration for photosynthesis.

*β_t_* is a function, ranging from one to zero that represents the soil moisture and the lower temperature effects on stomatal resistance and photosynthesis.
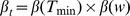


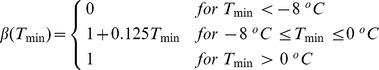


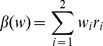


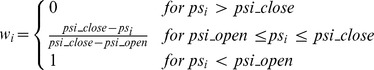
where *T_min_* is the daily minimum temperature; *w_i_* is the soil water stress of soil layer *i*; *ps_i_* is the soil water potential of soil layer *i,* which is estimated from the soil volume water content based on equations from Saxton and Rawls [40]; *r_i_* is the root fractions distributed in soil layer *i*; *psi_close* and *psi_open* are the plant functional specific tolerance of the soil water potential for stomata overall close and open. The water stress in plants is a function of soil water potential which ranges from 0 to 1. Under no water limitations, the soil water stress of soil layer *i* (*w_i_*) is equal to 1 where the soil water potential is at its maximum i.e., soil water potential when the stomata is opened (*psi_open*). Under frequent water stress, however, *wi* is calculated based on wilting point potential of specific plant functional types and depends on the balance between *psi_open* and *psi_close.*

Leaf stomatal resistance and leaf photosynthesis are coupled together through the following [37]–[39].
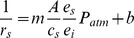
where *r_s_* is the leaf stomatal resistance, *m* is an empirical parameter, *A* is the leaf photosynthesis, *c_s_* is the leaf surface CO_2_ concentration, *e_s_* is the leaf surface vapor pressure, *e_i_* is the saturated vapor pressure inside leaf, *b* is the minimum stomata conductance with *A* = 0, and P*_atm_* is the atmospheric pressure. Together in the following equations:



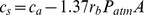


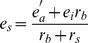
where *c_a_* is the atmospheric CO_2_ concentration, rb is the boundary resistance, *e_a_* is the vapor pressure of air, and stomatal resistance is the larger of the two roots of this quadratic [38].



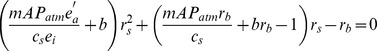



### 2.3 Modeling net primary productivity (NPP) in the DLEM

NPP is the net C gain by vegetation and equals the difference between GPP and plant respiration, which is calculated as:
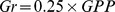


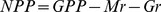



The DLEM estimates maintenance respiration (*Mr*, unit: gC m^−2^ day^−1^) and growth respiration (*Gr*, unit: gC m^−2^ day^−1^) as a function of assimilated C, surface air temperature and biomass nitrogen content. *Gr* is calculated by assuming that the fixed part of assimilated C will be used to construct new tissue (for turnover or plant growth). During these processes, 25% of assimilated C is supposed to be used as growth respiration [41]. Maintenance respiration is related to surface temperature and biomass nitrogen content. The following is used to calculate the maintenance respiration of leaves, sapwood, fine roots, and coarse roots:
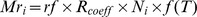



Where *i* denotes the C pool of different plant parts (leaf, sapwood, fine root, or coarse root); *Mr_i_* (gC m^−2^ day^−1^) is the maintenance respiration of different pools; *rf* is a parameter indicating growing phase, which is set at 0.5 for the non-growing season and 1.0 for the growing season; *R_coeff_* is a plant functional type-specific respiration coefficient; *N_i_* (gN m^−2^) is the nitrogen content of pool *i*; *f(T)* is the temperature factor and is calculated as follows:
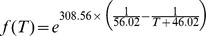



Where *T* is the daily average air temperature for modeling aboveground C pools such as leaves, sapwood, and heartwood or soil temperature for modeling belowground pools such as coarse roots and fine roots.

### 2.4 The effect of CO_2_ fertilization

In this study, we further quantified the effects of direct CO_2_ fertilization on terrestrial NPP by calculating the ‘beta’ (β) effect. β effect measures the strength of changes in terrestrial NPP in response to increasing CO_2_ concentration as follows:
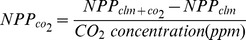



Where, *NPP_CO2_* is the relative contribution of direct CO_2_ fertilization on terrestrial NPP under the A2 and B1 scenarios, *NPP_clm+CO2_* is the terrestrial NPP under the climate plus CO_2_ experiment and *NPP_clm_* is the terrestrial NPP under the climate only experiment. CO_2_ concentration (ppm) is the concentration of atmospheric CO_2_ under the A2 and B1 scenarios.

### 2.5 Input datasets

**Figure 2:**
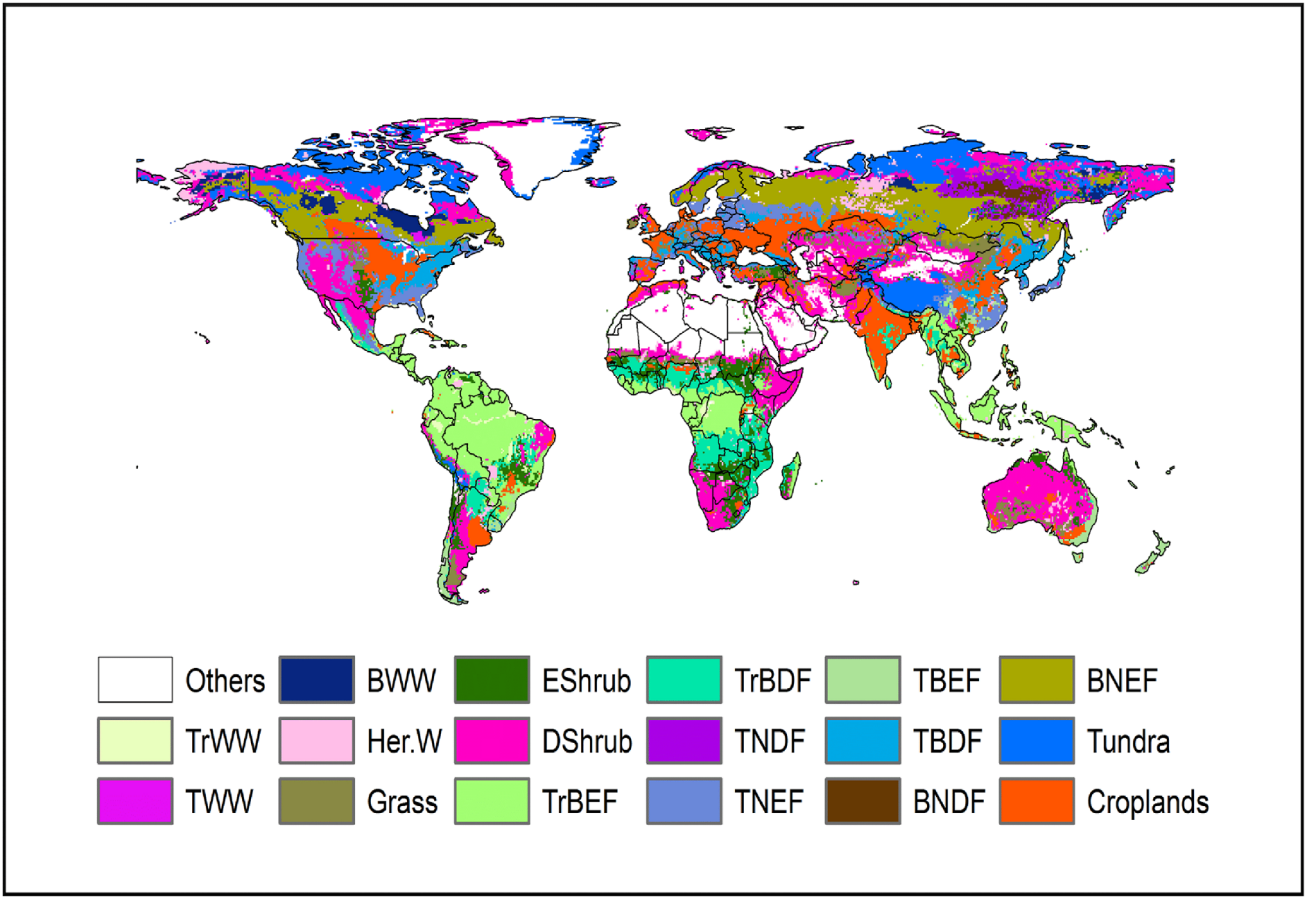
Contemporary vegetation map of the world as observed from the DLEM model for the year 2010. TrWW: Tropical Woody Wetlands; TWW: Temperate Woody Wetlands, BWW: Boreal Woody Wetlands, Her.W: Herbaceous Wetlands, EShrub: Evergreen Shrubland; DShrub: Deciduous Shrubland; TrBEF: Tropical Broadleaf Evergreen Forest; TrBDF: Tropical Broadleaf Deciduous Forest; TNDF: Temperate Needleleaf Deciduous Forest; TNEF: Temperate Needleleaf Evergreen Forest; TBEF: Temperate Broadleaf Evergreen Forest; TBDF: Temperate Broadleaf Deciduous Forest; BNDF: Boreal Needleleaf Deciduous Forest; BNEF: Boreal Needleleaf Evergreen Forest; Others: Desert & Ice.

The spatially-explicit data sets for driving the DLEM model include time series of daily climate, CO_2_ concentration, annual land cover and land use (LCLU), nitrogen deposition, tropospheric ozone, and land management practices (irrigation and nitrogen fertilizer use). Other ancillary data include river network, cropping system, soil property, and topography maps. Contemporary vegetation map include 18 plant functional types (Figure 2). Cropland and urban distribution datasets were developed by aggregating the 5-arc minute resolution HYDE v3.1 global cropland distribution data [42]. The vegetation map was developed based on global land-cover data derived from Landsat imageries [43], the National Land Cover Dataset 2000 (www.usgs.gov), and the global database of lakes, reservoirs, and wetlands [44]. The vegetation is transient and does not include any disturbance during the course of simulation. Half degree daily climate data (including average, maximum, minimum air temperature, precipitation, relative humidity, and shortwave radiation) were derived from newly available CRU-NCEP climate forcing data (1900–2009, 6-hour, half degree spatial resolution) [45]. The annual nitrogen deposition dataset for the historical period were based on Dentener [46]. Ozone AOT_40_ data sets were based on the global AOT_40_ index developed by Felzer et al. [47]. The gridded monthly CO_2_ concentration data were derived from Multi-scale Synthesis and Terrestrial Model Intercomparison Project (MSTIMP) (http://nacp.ornl.gov/MsTMIP.shtml). Consumption of nitrogen fertilizers from 1961 to 2008 were derived from country level Food and Agriculture Organization of the United Nations (FAO) statistic database (http://faostat.fao.org). We then calculated the annual fertilization rate (gN m^−2^) as the ratio of national fertilizer application amount to total cropland area in each country [48]. The contemporary irrigation map was developed based on LCLU data and global irrigatied fraction map [49], [50]. Long-term average climate datasets from 1900 to 1930 were used to represent the initial climate state in 1900.

**Figure 3:**
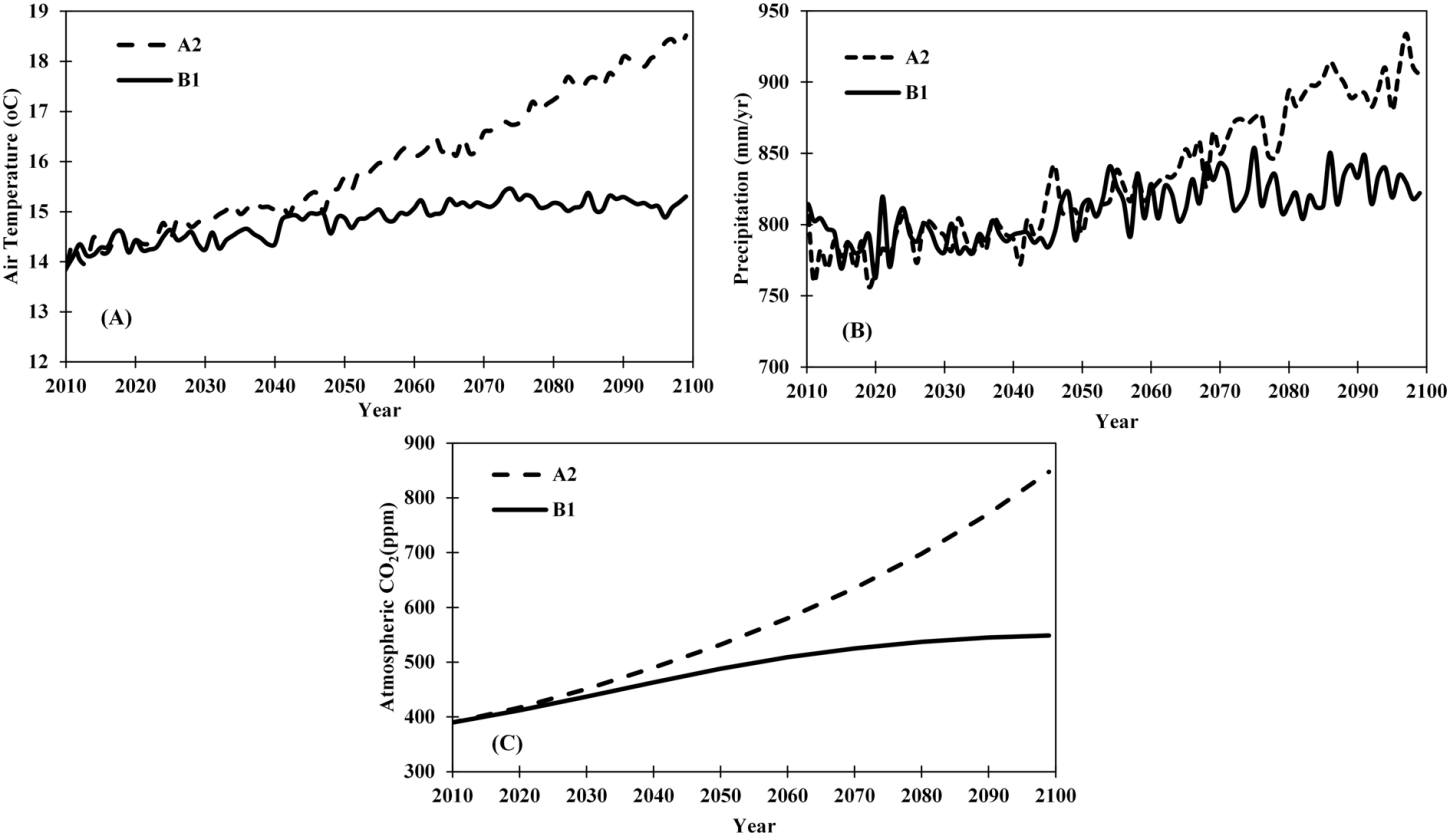
Input datasets used for driving the DLEM model based on CRUNCEP analysis. Temperature and precipitation change for A2 (A) and B1 (B) scenario and changes in CO_2_ concentration between A2 and B1 emission scenario.

**Figure 4:**
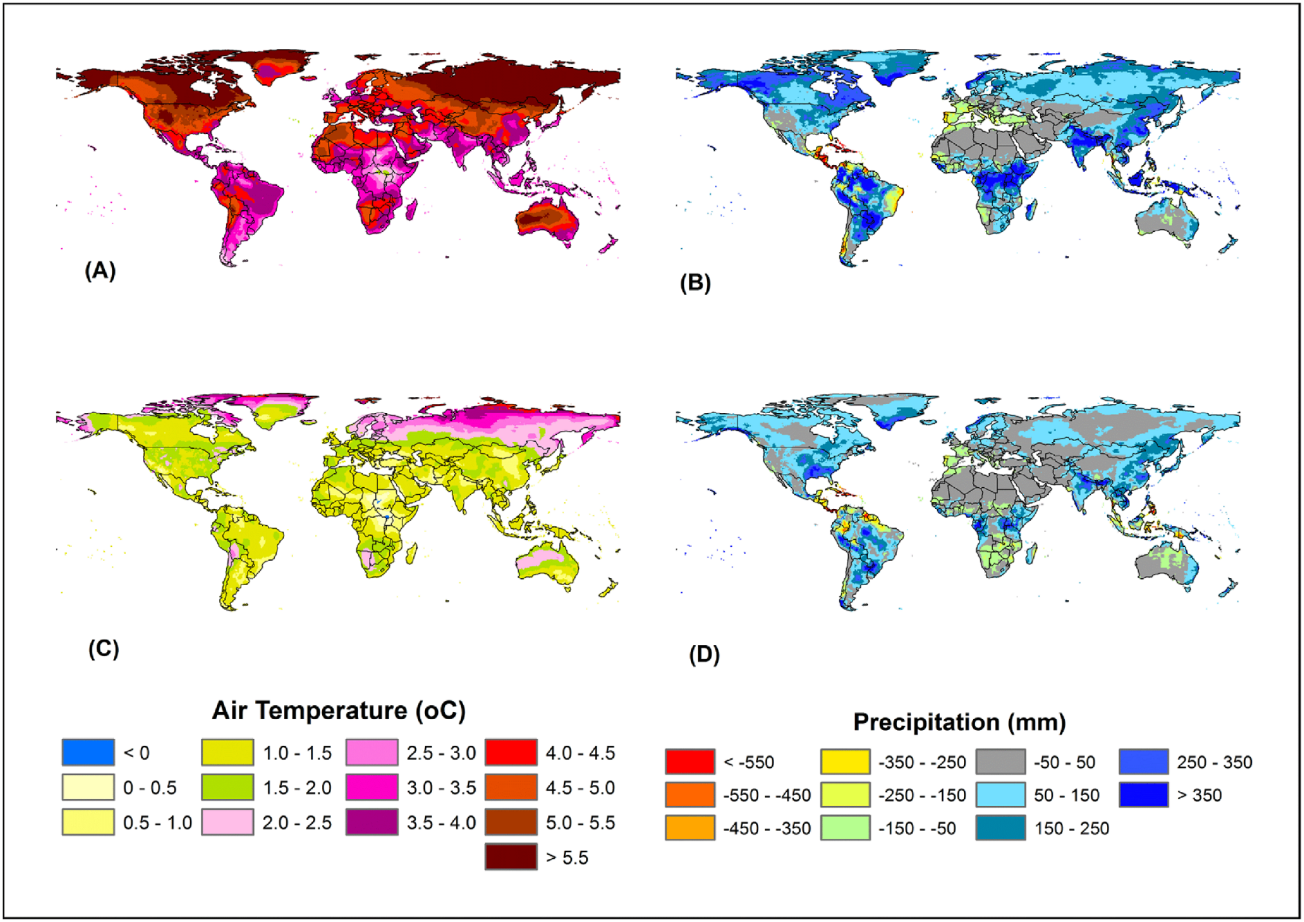
Spatial pattern of temperature and precipitation estimated as an average difference between 2099–2090 and 2000–2009: temperature (A) and precipitation (B) under A2 scenario and temperature (C) and precipitation (D) under B1 scenario.

For future projections, we used two IPCC emission secenarios (A2 and B1) datasets containing atmospheric CO_2_ concentration and climate (precipitation and temperature) from the Community Climate System Model (CCSM3) (Figure 3–4). The A2 scenario (high emission scenario) is characterized by rapid population growth and low per capita income, with regionally oriented economic development. The B1 scenario (low emission scenario) describes the same global population as the A2 storyline, but it is less materially intensive in its service and information, economic structure, with emerging clean and resource-efficient technology [6]. The climate datasets were downloaded from the World Climate Research Programme’s Coupled Model Intercomparison Project phase 3 (CMIP3) multi-model database (Meehl et al. [51]; www.engr.scu.edu/~emaurer/global_data). These datasets were downscaled as described by Maurer et al. [52] using the bias-correction/spatial downscaling method [53] to a 0.5 degree resolution, based on the 1950–1999 gridded observations of Adam and Lettenmaier [54]. For the future projections (2010–2099), we assumed that nitrogen deposition, ozone pollution, and LCLU remains unchanged from 2009.

Temperature and precipitation have been projected to increase substantially during 2010 to 2099 (Figure 3A–C) with large spatial variations under the A2 and B1 scenarios (Figure 4A–D). Under the A2 scenario, air temperature would increase by 4.6°C (Temperature = 0.008×Year; p-value<0.01), while precipitation would increase by 16.8% (Precipitation = 0.41×Year; p-value<0.01) by the 2090s compared to the 2000s. Similarly, under the B1 scenario, air temperature would increase by 1.5°C (Temperature = 0.007×Year; p-value<0.01), while precipitation would increase by 7.5% (Precipitation = 0.039×Year; p-value<0.01) during the 2090s compared to the 2000s. Across latitudes, the largest increase in surface air temperature (>5°C) would occur under the A2 scenario in mid- and high-latitude regions of the Northern Hemisphere, while the smallest increase would occur in low latitude regions. In the Southern Hemisphere, a large increase in surface air temperature (>6°C) would occur in parts of Australia. The largest increase in precipitation would occur in high-latitude regions under the A2 and B1 scenarios, while large variations in mid- and high-latitude regions. For instance, there would be no change in precipitation in Africa, Southwestern US, Northwestern China and Australia under both scenarios, while precipitation would increase by >350 mm and >250–350 mm in monsoon Asia under the A2 and B1 scenarios, respectively. In addition, large variation in total precipitation between the Southern and the Northern Hemisphere have been observed due to physical distribution of more landmass resulting in a greater thermal effect in the Northern Hemisphere than in the Southern Hemisphere [55].

### 2.6 Model parameterization, calibration and evaluation

**Figure 5:**
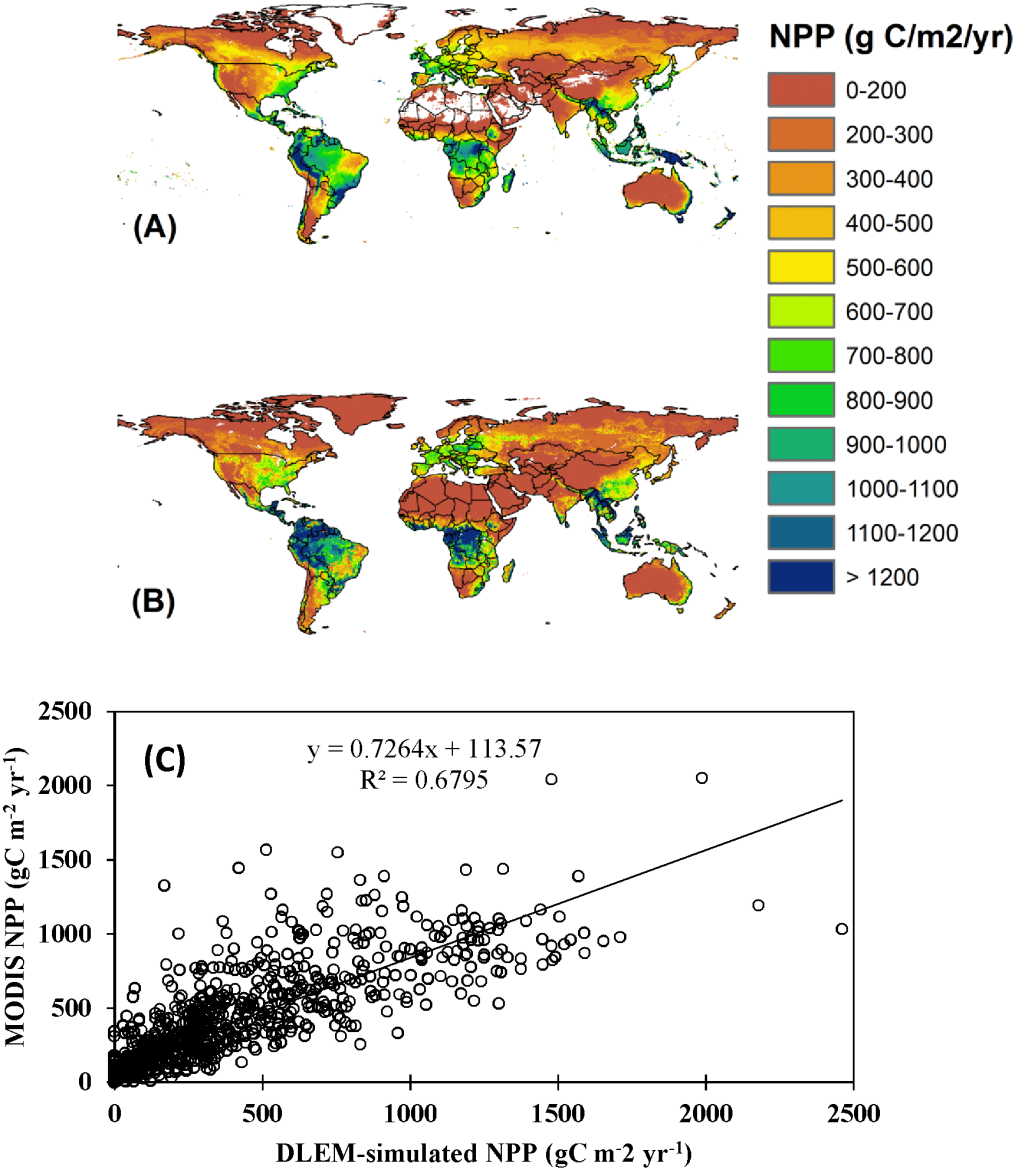
Spatial patterns of MODIS-NPP (A) and DLEM-simulated NPP (B) during 2000–2009 and comparison of the DLEM-simulated NPP with MODIS-NPP (C) for 6000 randomly selected grids.

The DLEM has been parameterized and applied across several regional and continental studies including Asia [31], [33], [56]–[60], the United States [22], [34], [61] and North America [62], [63] using long-term observational data for all defined plant functional types. The calibrated parameter values have been used to drive the model for specific plant functional types (Figure 5). In this study, we compared DLEM-simulated global estimates of terrestrial NPP with MODIS NPP to evaluate model performance during 2000–2009. We first evaluated DLEM performance at the global level in simulating spatial pattern of terrestrial NPP by comparing DLEM-simulated NPP with MODIS NPP (Figure 5). We then carried out a grid-to-grid comparison of DLEM-simulated NPP with MODIS product by randomly selecting 6000 grids from MODIS product (10% of the total sampling units). For bare land such as part of Africa, China and Mongolia (Taklamakan desert in West China, Gobi desert in Mongolia), there is no NPP data available from MODIS and we excluded those areas from analysis. At the global scale, the spatial pattern of DLEM-simulated NPP is consistent with that of MODIS NPP (Figure 5A–B). In addition, we found a good agreement between MODIS and DLEM-simulated NPP for randomly selected 6000 grid points (Figure 5C).The fitted line between DLEM-simulated and MODIS derived NPP had a slope of 0.73 and a high correlation coefficient (R^2^ = 0.68). Our model evaluation for the effect of CO_2_ fertilization is available in Lu et al. [60].

### 2.7 Experimental design and model implementation

We designed 2×2 factorial simulation experiments (2 simulations×2 scenarios). The two simulation experiments include: (1) climate change only: only climate changes with time and other environmental factors are held constant during the study period (2010–2099); and (2) climate plus CO_2_: both climate and atmospheric CO_2_ concentration change during the study period while other environmental factors including LCLU, nitrogen deposition, and tropospheric ozone are held constant at 2009 leve1s. The A2 and B1 climate scenarios were used to drive these two simulations.

The model simulation follows three important stages: an equilibrium simulation stage, a 3000-year spin-up stage, and a transient simulation stage. The model simulation begins with an equilibrium run with long-term average climate data for the period 1901–1930, with the 1900 levels of atmospheric CO_2_ concentration, nitrogen deposition, and LCLU map to develop simulation baselines for C, nitrogen, and water pools. However, the tropospheric ozone data is kept at the 1935 level during the equilibrium. The simulation baseline (equilibrium) is approached when the net C exchange between the atmosphere and terrestrial ecosystems is less than 0.1 gC m^−2^, the change in soil water pool is less than 0.1 mm, and the difference in soil mineral nitrogen content and nitrogen uptake is less than 0.1 gN m^−2^ among consecutive years. After the equilibrium run, a 3000-year spin up is carried out using transient climate data and LCLU distribution in the 1900 to eliminate system fluctuations caused by simulation mode shift from equilibrium to transient mode. Finally, a transient simulation is set up, driven by changes in climate, atmospheric chemistry (nitrogen deposition, tropospheric ozone, and atmospheric CO_2_ concentration), and LCLU distribution during 1900–2009. In this study, we focused our analysis on global terrestrial NPP during two time periods: 2000–2009 and 2010–2099. For the first period (2000–2009), the simulated results (NPP_2000s_) represent contemporary patterns of global terrestrial NPP. For the second period (2010–2099), simulated results (NPP_2090s_) reflect the evolution of terrestrial NPP by the end of the 21^st^ century. The difference between the two decadal mean NPP (NPP_2090s_ - NPP_2000s_) indicates the overall effects of climate change and CO_2_ increase on terrestrial ecosystems in the 21^st^ century.

## Results

### 3.1 Terrestrial NPP in the first decade of 21^st^ century

**Figure 6:**
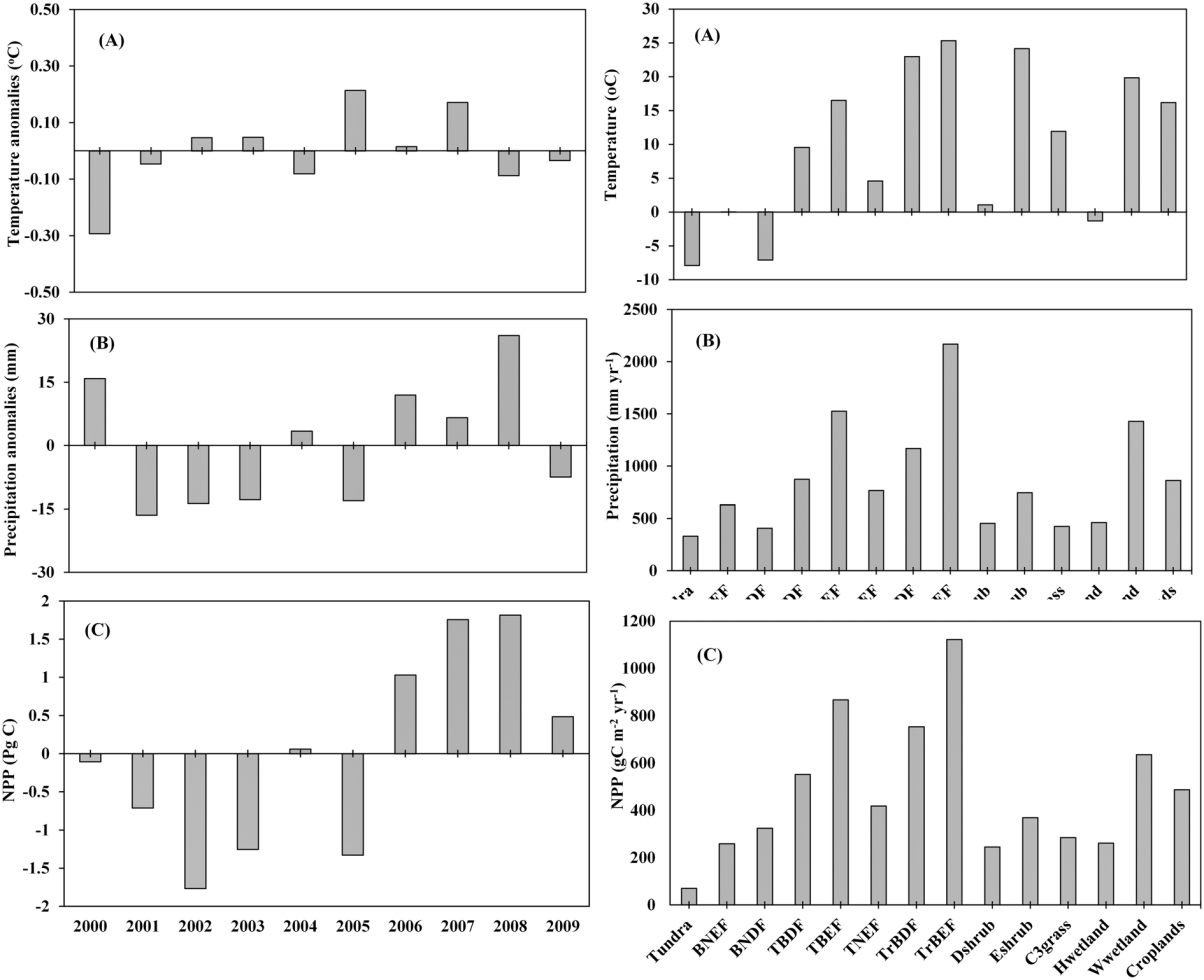
Effect of inter-annual variation in precipitation and temperature on global net primary productivity during the contemporary period (2000–2009) (left panel) and changes in mean annual NPP of major biomes as a function of temperature and precipitation (right panel). Left Panel: Mean annual temperature anomalies (a), annual precipitation anomalies (b), and net primary productivity (c) and right panel: average (2000–2009) temperature (a) average precipitation (b) and average net primary productivity (c).

The DLEM simulation results show a global terrestrial NPP of about 54.57 (52.8–56.4) PgC yr^−1^ during the first decade of the 21^st^ century, with substantial inter-annual variations due to precipitation (R^2^ = 0.63; P<0.01) (Figure 6; left panel). In a specific year, drought or wet climate could substantially alter the magnitude of global terrestrial NPP. For instance, the dry year of 2005 resulted in a decline in global terrestrial NPP by 1.33 Pg C, while the wet year of 2008 increased global terrestrial NPP by 1.82 Pg C compared to the decadal mean (2000–2009). In addition, there are substantial variation in contemporary productivity across different biomes, with the highest NPP of 1122.43 gC m^−2^ yr^−1^ for tropical broadleaf evergreen forest and the lowest NPP of 70.27 gC m^−2^ for tundra vegetation (Figure 6; right panel). It should be noted that boreal needleleaf deciduous forests have experienced fast increase in NPP by 7.42 gC m^−2^ yr^−1^ (p-value<0.05) during 2000–2009, due to substantial warming in the high latitudes [64]. To examine whether the contemporary trend will continue, we further projected climate change effects on ecosystem productivity during the rest of the century (2010–2099).

### 3.2 Changes in terrestrial NPP induced by climate change and increasing atmospheric CO_2_ during 2010–2099

#### 3.2.1.Temporal responses of terrestrial NPP to climate change

**Figure 7:**
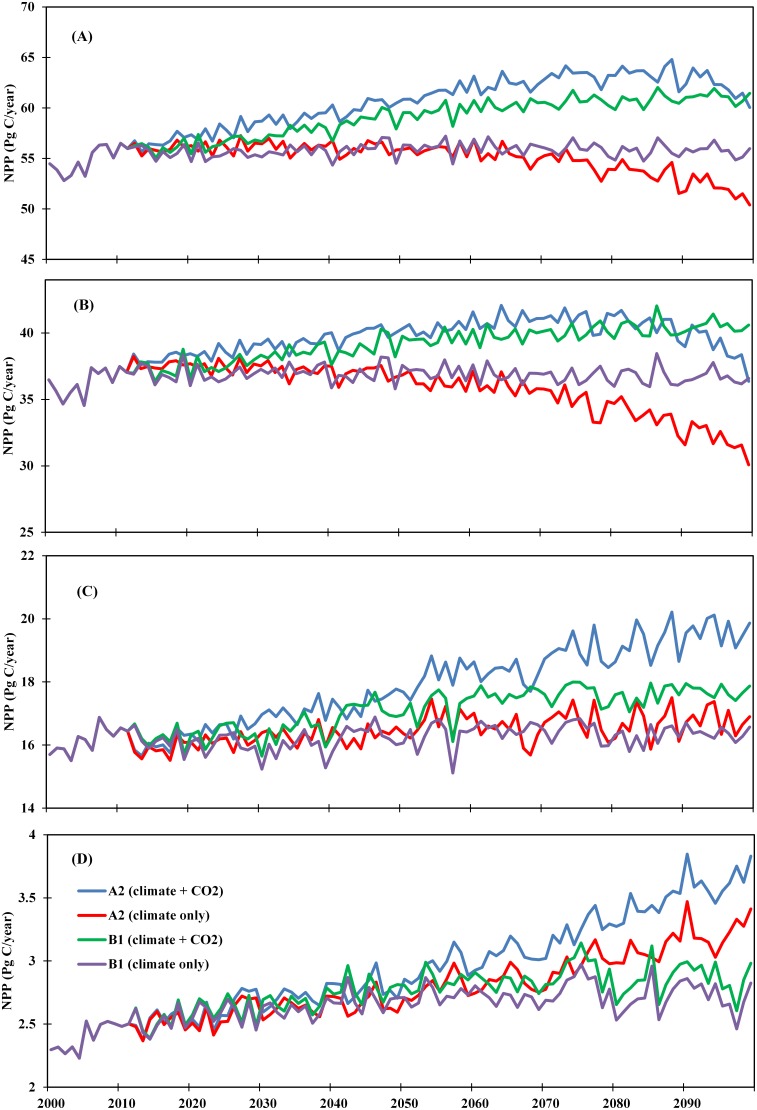
Temporal pattern of change in terrestrial NPP: Global (A), low-latitude (B), mid-latitude (C) and high-latitude (D) as a function of climate and increasing atmospheric CO_2_ under A2 and B1.

**Figure 8:**
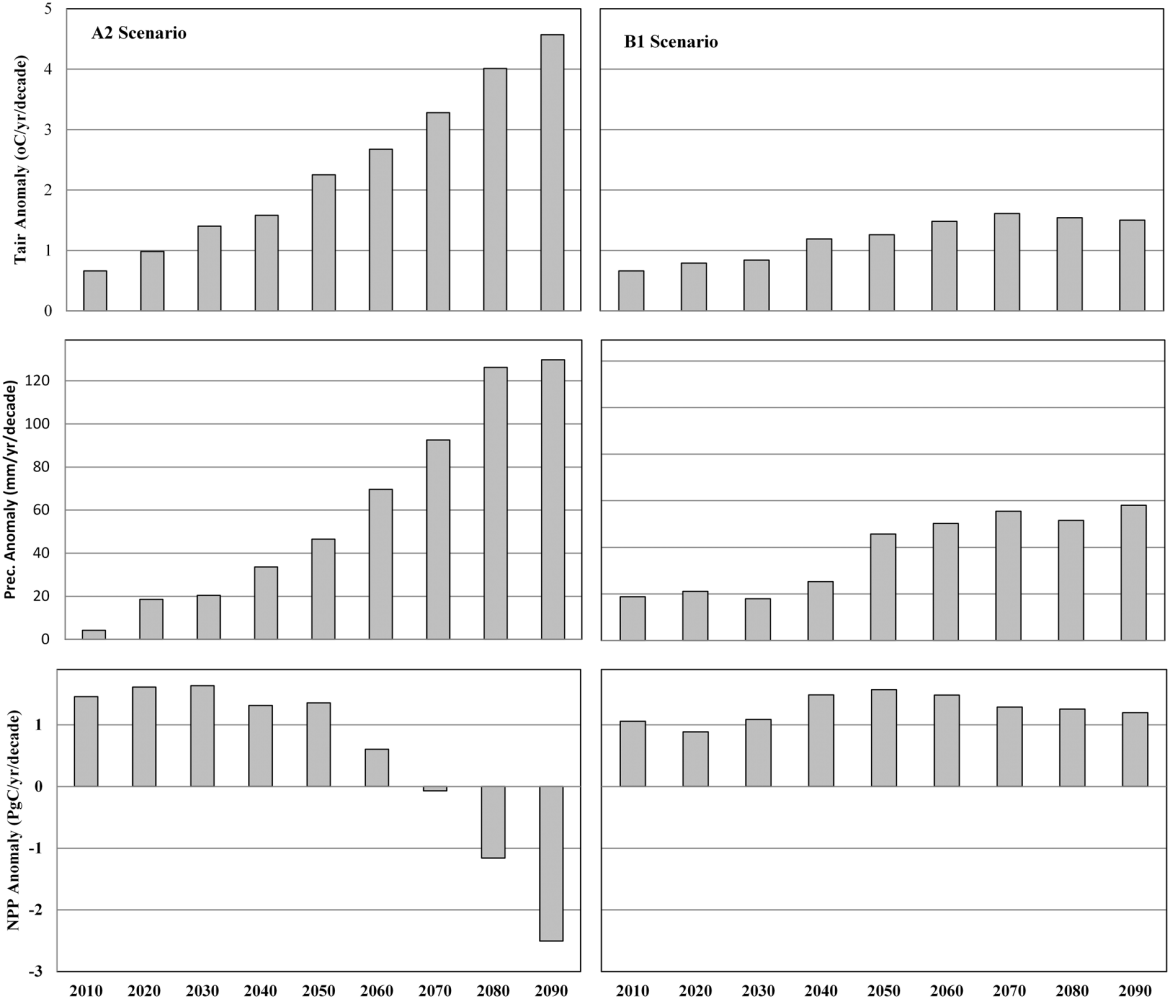
Effect of temperature and precipitation on global net primary productivity during the rest of the 21^st^ century (2010–2099) under A2 (left panel) and B1 (right panel) climate change scenarios.

Our DLEM simulations show that climate change would increase terrestrial NPP by 3.0% until the 2030s under the A2 scenario and by 2.7% until the 2060s under the B1 scenario (Figure 7A; Table 1), but there would be a declining trend afterwards. Climate change under the A2 scenario would result in an overall decline in terrestrial NPP by 2.51 PgC (4.6%) in the 2090s compared to the 2000s (Figure 8; left panel). The B1 scenario shows an increasing trend with the highest increase in NPP by 1.57 PgC in the 2050s; however, NPP would level off after the 2050s with an overall increase by 1.2 PgC (2.2%) in the 2090s compared to the 2000s (Figure 8; right panel). Under both A2 and B1 scenarios, we found a levelling off of NPP when temperature reaches ∼15°C (i.e. a 1.5°C rise compared to the contemporary period (2000–2009) global mean temperature). Interestingly, NPP would decline below the contemporary level after the 2060s when temperature exceeds approximately 16.5°C under the A2 scenario (Figure 8; left panel). Global terrestrial NPP, however, shows a complex temporal response to changes in precipitation during the 21^st^ century. NPP increases with increasing precipitation through the 21^st^ century under the B1 scenario (P-value<0.01) while under the A2 scenario, it increases until the 2060s (P-value<0.2).

**Table 1:** Decadal changes in global terrestrial net primary production (NPP) and across low-, mid-, and high-latitude regions under A2 and B1 scenario for climate and climate plus CO_2_ experiments.

Global NPP (PgC yr^−1^)									
2000s	54.57								
	A2 (Climate Only)		A2 (Climate + CO_2_)			B1(Climate Only)			B1 (Climate + CO_2_)
**2030s**	56.20(3.0%)		59.13(8.4%)			55.66(2.0%)			57.68(5.7%)
**2060s**	55.17(1.1%)		62.30(14.2%)			56.05(2.7%)			60.27(10.5%)
**2090s**	52.06(−4.6%)		62.18(13.9%)			55.77(2.2%)			61.12(12.0%)
**Low Latitude NPP (PgC yr^−1^)**									
**2000s**		**36.09**							
		**A2 (Climate Only)**		**A2 (Climate + CO_2_)**	**B1(Climate Only)**			**B1 (Climate + CO_2_)**	
**2030s**		37.24(3.2%)		39.32(8.9%)	37.10(2.8%)			38.51(6.7%)	
**2060s**		35.90(−0.5%)		40.96(13.5%)	36.83(2.0%)			39.83(10.4%)	
**2090s**		32.02(−11.3%)		38.92(7.8%)	36.70(1.7%)			40.57(12.4%)	
**Middle Latitude NPP (PgC yr^−1^)**									
**2000s**		**16.09**							
		**A2 (Climate Only)**		**A2 (Climate + CO_2_)**	**B1(Climate Only)**		**B1 (Climate + CO_2_)**		
**2030s**		16.32(1.4%)		17.08(6.2%)	15.94(−0.9%)		16.49(2.5%)		
**2060s**		16.43(2.1%)		18.28(13.6%)	16.53(2.7%)		17.62(9.5%)		
**2090s**		16.83(4.6%)		19.64(22.0%)	16.36(1.6%)		17.69(9.9%)		
**High Laitude NPP (PgC yr^−1^)**									
**2000s**		**2.39**							
		**A2 (Climate Only)**		**A2 (Climate + CO_2_)**	**B1(Climate Only)**		**B1 (Climate + CO_2_)**		
**2030s**		2.65(10.9%)		2.73(14.5%)	2.62(9.7%)		2.68(12.4%)		
**2060s**		2.84(19.1%)		3.06(28.1%)	2.70(13.0%)		2.82(18.2%)		
**2090s**		3.21(34.8%)		3.62(51.9%)	2.71(13.6%)		2.86(19.9%)		

#### 3.2.2. Temporal response of terrestrial NPP to climate change and increasing CO_2_ concentration

Climate change coupled with increasing atmospheric CO_2_ concentration would result in higher global terrestrial NPP than climate change alone under both the A2 and B1 scenarios (Figure 7A). The largest increase in terrestrial NPP would occur under the A2 scenario with an overall increase in the 2060s by 7.73 PgC (14.2%) compared to the 2000s; however, NPP would show a declining trend after the 2060s with a net decrease of 0.12 PgC (compared to the 2060s) in the 2090s. The B1 scenario shows an overall increasing trend through the 21^st^ century with the largest increase in terrestrial NPP by 6.55 PgC (12.0%) (Table 1).

### 3.3 Spatial variation of terrestrial NPP induced by climate change and increasing atmospheric CO_2_ during 2010–2099

#### 3.3.1. Latitudinal and spatial responses of terrestrial NPP to climate change

Our results show substantial variation in climate-induced NPP change along latitudes during the 21^st^ century (Figure 7B–D; Table 1). The magnitude of terrestrial NPP would be highest in low latitude regions (30.1–42.1 PgC yr^−1^), and lowest in high latitude regions (2.23–3.85 PgC yr^−1^). Compared to the 2000 s, the A2 climate would increase terrestrial NPP in mid- and high-latitudes by 4.6% and 34.8%, respectively, but would decrease terrestrial NPP in low latitude by 11.3% in the 2090s. The B1 climate scenario shows similar temporal pattern for mid- and high-latitidue regions with an increase in terrestrial NPP by 1.6% and 13.6%, respectively. The low latitude regions, however, show a declining trend with an increase in terrestrial NPP by 2.0% in the 2060s and 1.7% in the 2090s when compared to the 2000s. Under the A2 climate-only scenario, terrestrial NPP would increase in high latitude region by 0.82 PgC while decrease in low latitude terrestrial NPP by 4.07 PgC during the 2090s compared to the 2000s.

**Figure 9:**
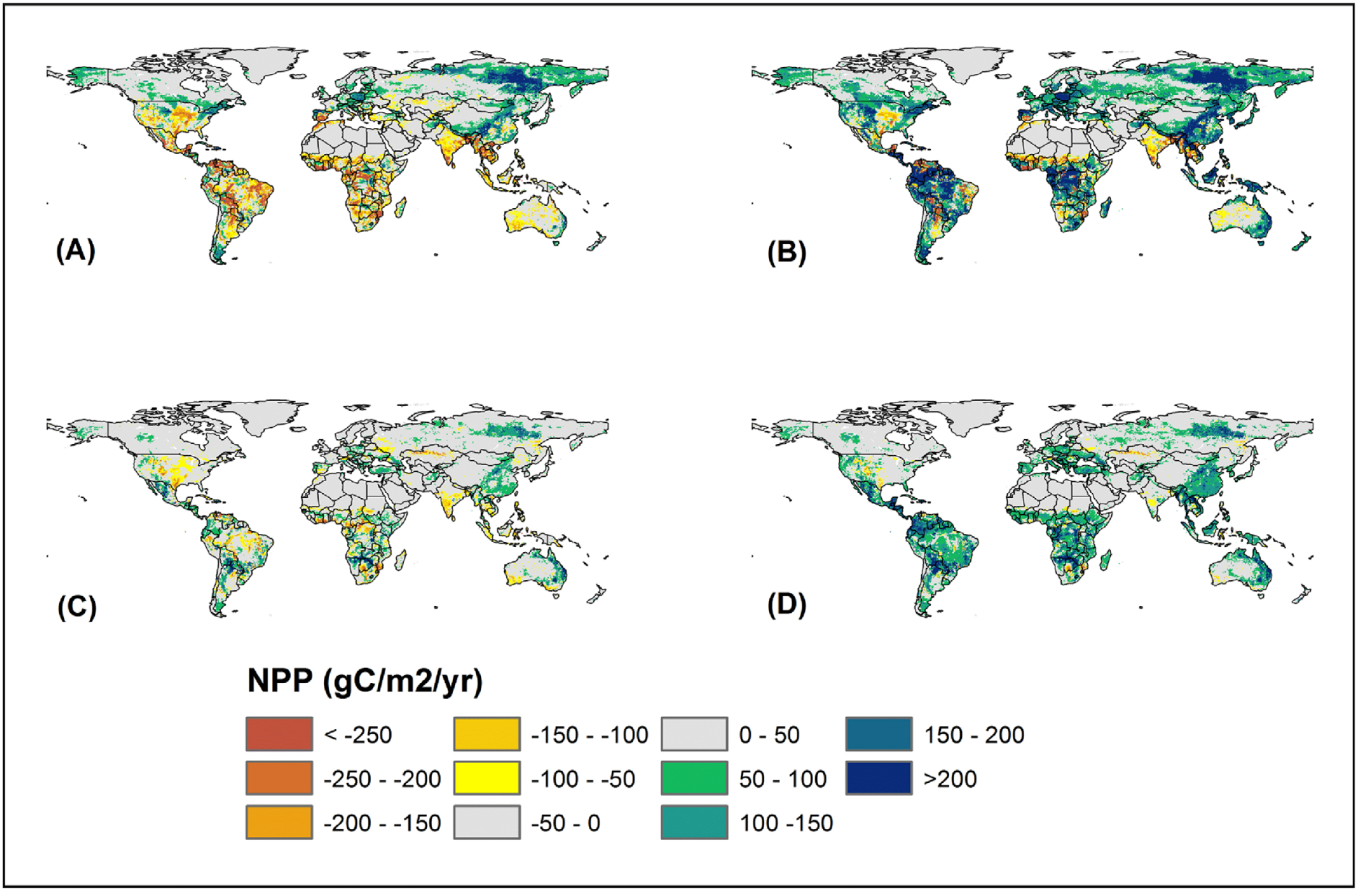
Spatial variation in terrestrial NPP as influenced by climate-only and climate with CO_2_. Climate only (A) and climate with CO_2_ (B) under A2 scenario, and climate only (C) and climate plus CO_2_ (D) under B1 scenario.

**Figure 10:**
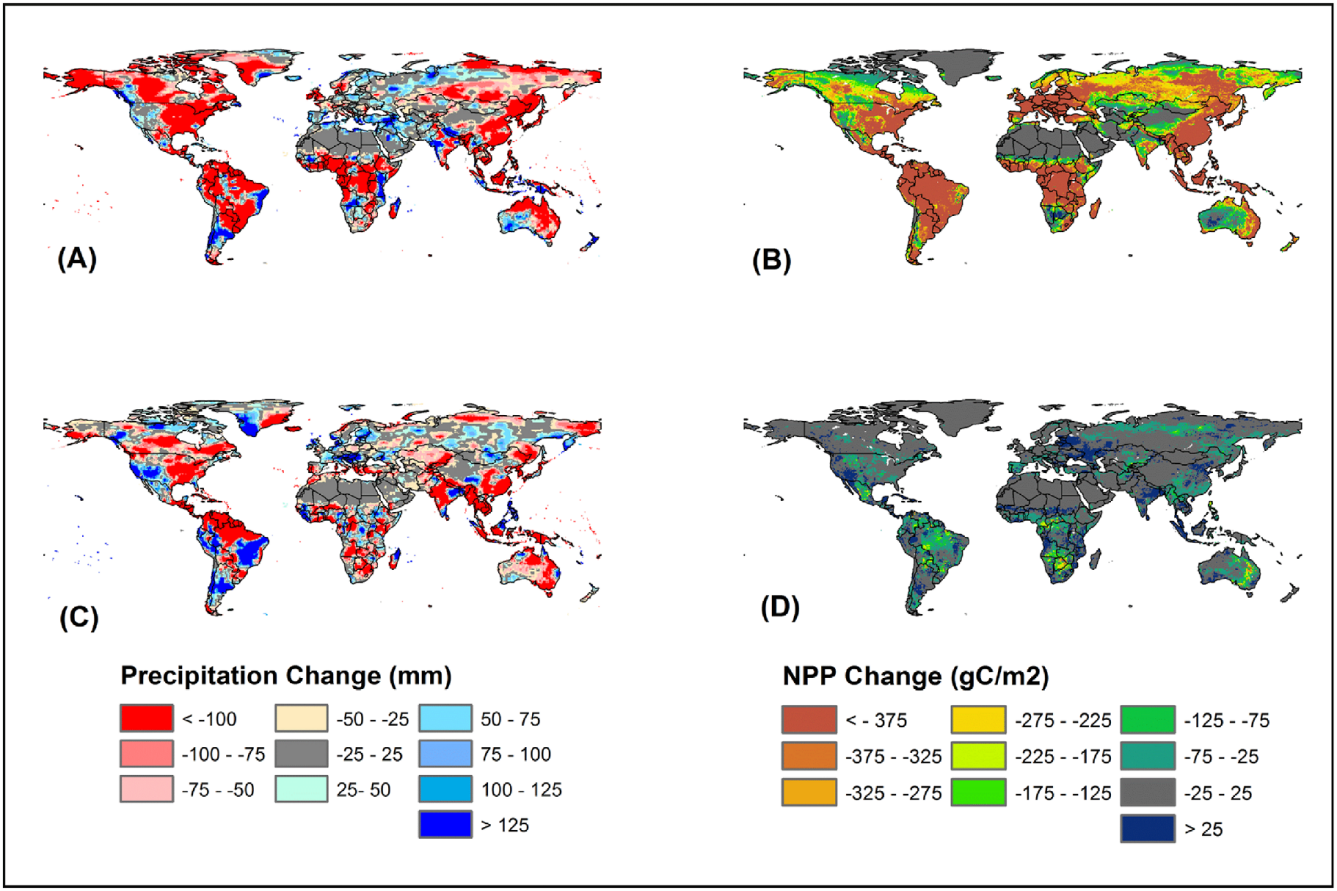
Spatial variation in precipitation and NPP estimated as a difference between dry year and long term (2000–2099) mean: precipitation difference between 2019 and long term mean (A) for A2 scenario, NPP difference between 2019 and long term mean (B) for A2 scenario climate-only simulation, precipitation difference between 2020 and long term mean (C) for B1 scenario and NPP difference between 2020 and long term mean (D) for B1 climate-only simulation.

Our climate-only experiment shows an increase in terrestrial NPP by 50–100 gC m^−2^ yr^−1^ in boreal and arctic regions (Figure 9) under both the A2 and B1 scenarios. However, the largest decline in terrestrial NPP would occur in tropical regions (>250 gC m^−2^ yr^−1^) such as parts of South America, Africa, South Asia and Australia under the A2 scenario due to rapidly warming temperature and likely drought events. The climate-only experiment under the B1 scenario, however, shows no substantial decrease in terrestrial NPP in tropical regions. Our results further show that in a particular year, drought or rainfall deficits combined with increasing temperature can potentially reduce terrestrial NPP. Compared to the average precipitation of the 21^st^ century, terrestrial ecosystems experienced the largest reduction in global precipitation (Figure 10) of about 68.97 and 43.07 mm yr^−1^ in 2019 and 2020 under the A2 and B1 scenario, respectively. This annual precipitation deficit resulted in a reduction in global terrestrial NPP by 375 gC m^−2^ in the tropics and low latitude regions (Figure 10).

#### 3.3.2. Latitudinal and spatial responses of terrestrial NPP to climate change and increasing CO_2_ concentration

Climate plus CO_2_ experiment under both the A2 and B1 scenarios show substantial difference in the magnitude and temporal pattern of terrestrial NPP along latitudes (Figure 7B–D). The A2 climate plus CO_2_ experiment shows a substantial increase in terrestrial NPP in low latitude regions until the 2060s where NPP would increase by 4.87 PgC which is equivalent to an increase of 13.5%. However, terrestrial NPP would start to decline in the 2090s (Table 1). In mid- and high-latitude regions, terrestrial NPP would continue to increase through the 21^st^ century, with an increase of about 22.0% and 51.9%, respectively by the end of the century (2090s vs. 2000s). The climate plus CO_2_ experiment under the B1 scenario shows an increasing trend in terrestrial NPP across all latitudes where NPP would increase by 12.4%, 9.9%, and 19.9% in low-, mid-, and high-latitude regions, respectively. During the 2090 s, the largest magnitude (4.48 PgC) of increase in terrestrial NPP would occur in low latitude regions under the B1 scenario, while the largest rate (51.9%) of increase would occur in high latitude regions, under the A2 scenario.

Large increase in terrestrial NPP would occur in tropical regions especially in central and southern Africa, and the Amazon basin under the A2 scenario, while modest increase under the B1 scenario (Figure 9B, 9D). The A2 scenario shows large increase in terrestrial NPP by >200 gC m^−2^ yr^−1^, while the B1 scenario shows an increase of 100–200 gC m^−2^ yr^−1^ in tropical regions. Large increase in NPP in the tropical regions due to increasing atmospheric CO_2_ concentration is primarily because DLEM uses a Farquhar model that shows a higher NPP enhancement at high temperatures under elevated CO_2_. However, the mid- and high-latitude regions show lower NPP enhancement in response to increasing CO_2_ concentration possibly because of nitrogen limitations.

### 3.4 Biome variation of terrestrial NPP response to climate change and increasing CO_2_ during 2010–2099

#### 3.4.1. Biome NPP response to climate change

Our results show large variation in the response of various biomes to future climate change (Table 2). The A2 climate scenario would result in a NPP decrease for major biomes such as tropical broadleaf deciduous and tropical evergreen forest, evergreen shrubs, grasses, woody wetland and croplands in the 2090s. The largest percent decline would occur in tropical broadleaf evergreen forest where NPP would decrease by 104.0 gC m^−2^, equivalent to a decrease of 9.3% compared to the contemporary NPP. The largest percent increase under the A2 scenario would occur in tundra and boreal needleleaf deciduous forest by 41.5 gC m^−2^ and 176 gC m^−2^ equivalent to an increase of 59.1% and 54.3%; respectively. The B1 climate scenario, however, shows no substantial change in terrestrial NPP across different biomes compared to the A2 scenario. We found the largest percent decline in NPP in woody wetland by 2.4%, and the largest percent increase in NPP in boreal needleleaf deciduous forest by 16.6% in the climate-only simulation under the B1 scenario. Croplands, in particular, show a decline in NPP due to climate variability under both the A2 and B1 scenarios with the largest decline of 9.9% under the A2 scenario.

**Table 2:** Decadal mean of terrestrial net primary production (NPP) in the contemporary period (2000–2009) and change in NPP between the 2090s and the 2000s among major biomes.

	Decadal Mean	A2B1	
	2000s	Net Change (% change)			
PFTs	(gC m^−2^ yr^−1^)	climate only	Climate + CO_2_	climate only	Climate + CO_2_
Tundra	70.27	41.5 (59.1)	55.1 (78.4)	8.2 (11.7)	13.0 (18.5)
BNEF	258.64	2.4 (0.9)	34.3 (13.3)	4.1 (1.6)	19.3 (7.5)
BNDF	324.14	176.0 (54.3)	257.6 (79.5)	53.8 (16.6)	74.0 (22.8)
TBDF	551.64	48.7 (8.8)	116.2 (21.1)	20.6 (3.7)	55.0 (10.0)
TBEF	867.63	18.2 (2.1)	187.2 (21.6)	15.8 (1.8)	78.8 (9.1)
TNEF	418.12	25.6 (6.1)	87.3 (20.9)	8.9 (2.1)	36.1 (8.6)
TrBDF	753.62	−76.1 (−10.1)	56.1 (7.4)	35.4 (4.7)	105.1(13.9)
TrBEF	1122.43	−104.0 (−9.3)	129.6 (11.5)	−1.6 (−0.1)	106.1 (9.4)
Deciduous Shrub	245.39	38.8 (15.8)	85.6 (34.9)	20.7 (8.4)	40.1 (16.3)
Evergreen Shrub	369.19	−55.8 (−15.1)	22.2 (6.0)	26.1 (7.1)	77.0 (20.9)
C3 grass	284.8	−0.6 (−0.2)	65.0 (22.8)	9.9 (3.5)	40.2 (14.1)
Herbaceous wetland	261.8	36.3 (13.9)	79.5 (30.4)	14.1 (5.4)	33.6 (12.8)
Woody wetland	635.90	−86.2 (−13.5)	29.5 (4.6)	−15.8 (−2.4)	52.3 (8.2)
Cropland	487.02	−48.2 (−9.9)	21.6 (4.4)	−4.3 (−0.9)	45.9 (9.4)

**Note:** BNEF, Boreal Needleleaf Evergreen Forest; BNDF, Boreal Needleleaf Deciduous Forest; TBDF, Temperate Broadleaf Deciduous Forest; TBEF, Temperate Broadleaf Evergreen Forest; TNEF, Temperate Needleleaf Evergreen Forest; TrBDF, Tropical Broadleaf Deciduous Forest; TrBEF, Tropical Broadleaf Evergreen Forest.

Numbers in parenthesis represents percentage change between the 2090s and the 2000s.

#### 3.4.2 Biome NPP response to climate change and increasing CO_2_ concentration during 2010–2099

Climate change and increasing atmospheric CO_2_ concentration would result in an overall increase in terrestrial NPP across all biomes through the 21^st^ century under both the A2 and B1 scenarios (Table 2). The strength of CO_2_ fertilization effect would be largest in tundra and boreal needleleaf deciduous forest under the A2 scenario where NPP would increase by 55.1 gC m^−2^ and 257.6 gC m^−2^ equivalent to an increase of 78.4% and 79.5%; respectively. Simulations with climate change and increasing atmospheric CO_2_ would result in an increase in tropical broadleaf evergreen forest NPP by 129.6 gC m^−2^ (which decreases with climate alone), equivalent to a percent increase of 11.5%, indicating that CO_2_ ferilization could have a substantial effect on terrestrial NPP. The B1 scenario, however, shows a relatively small increase in terrestrial NPP (7.5–22.8%) across all biomes. Interestingly, croplands show the largest increase in NPP by 45.9 gC m^−2^ (9.4%) under B1 scenario when climate change and increasing atmospheric CO_2_ concentration are included in an experiment. This increase is higher than that under the A2 scenario when climate change is coupled with increasing atmospheric CO_2_ concentration.

### 3.5 The CO_2_ fertilization effects on terrestrial NPP

**Figure 11:**
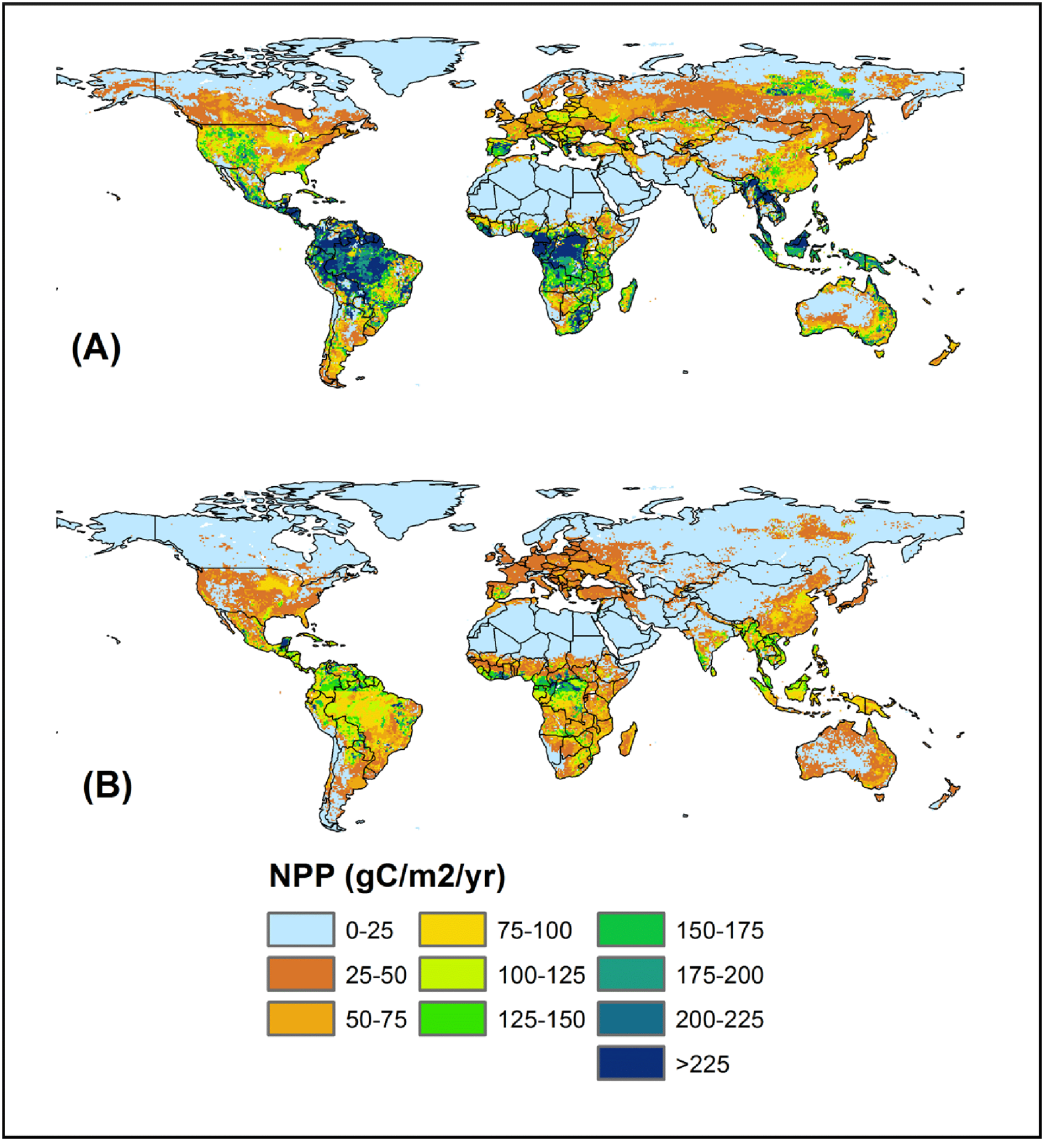
Contribution of increasing atmospheric CO_2_ concentration to NPP during the 2090s calculated as a difference between climate plus CO_2_ and climate-only experiments: A2 scenario (A) and B1 scenario (B).

Our results show that terrestrial NPP during the 2090s would increase due to increasing atmospheric CO_2_ across the globe, but the magnitude of increase varied substantially across different latitudes (Figure 11A–B). The largest increase in terrestrial NPP due to increasing atmospheric CO_2_ would be prevalent in the tropical regions where NPP would increase by >225 gC m^−2^ under the A2 scenario and 75–100 gC m^−2^ under the B1 scenario. A closer look at the effect of atmospheric CO_2_ elevation under the A2 scenario shows large increase in global NPP of about 10.1 PgC, equivalent to an increase of 18.5% during the 2090s compared to the 2000s.

**Figure 12:**
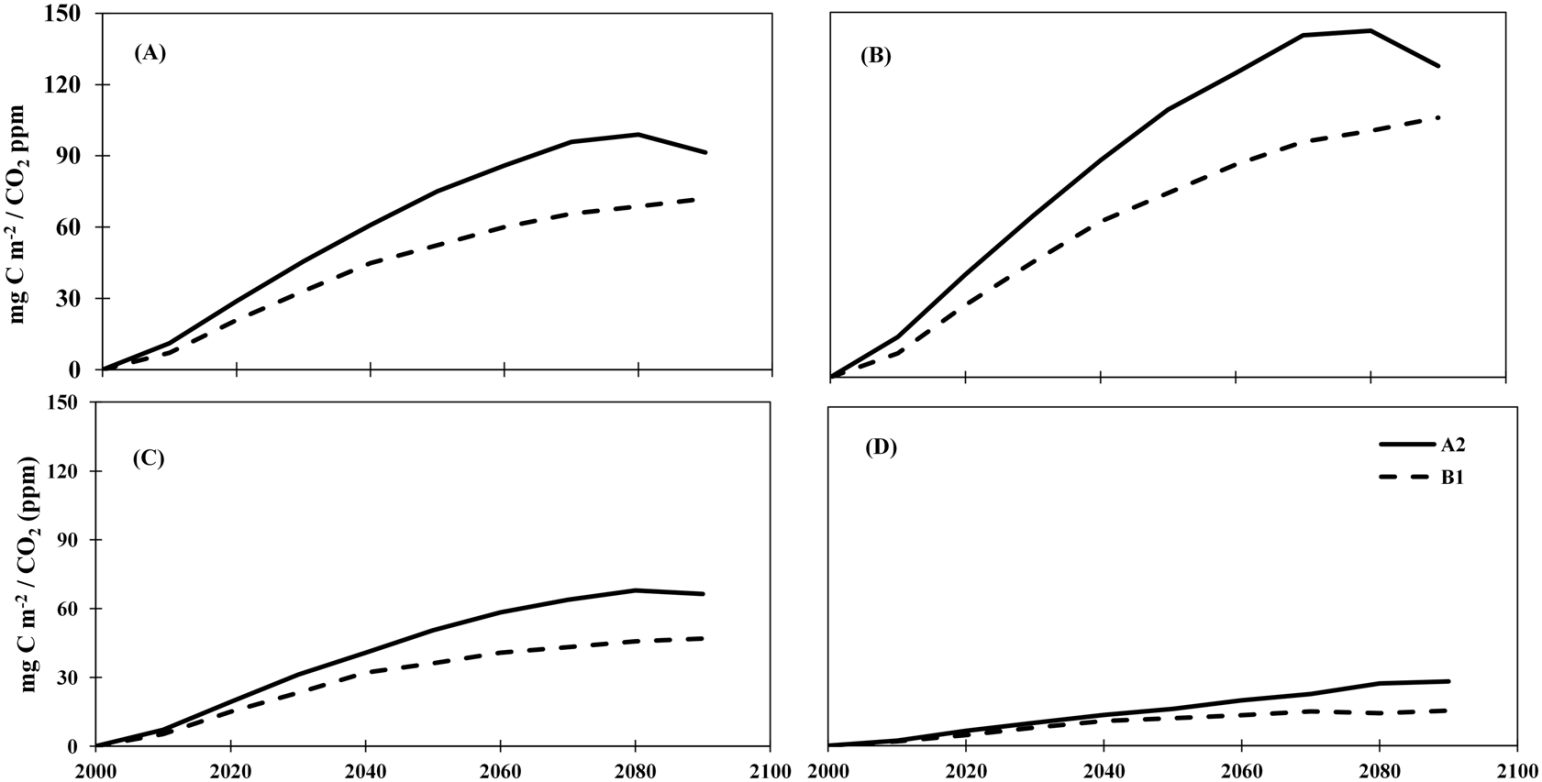
The effect of CO_2_ ferilization on terrestrial NPP across the globe (A), low-latitude (B), mid-latitude (C), and high-latitude (D) under A2 and B1 scenarios. For each unit of CO_2_ (ppm), the A2 scenario show a highest rate of increase in NPP (mgC m^−2^) compared to B1 scenario.

We further calculated the ‘beta’ (β) effect that measures the strength of changes in terrestrial NPP in response to increasing atmospheric CO_2_ concentration to examine the relative contribution of direct CO_2_ fertilization on terrestrial NPP (Figure 12). By the end of 21^st^ century, large increase in global terrestrial NPP would occur under the A2 scenario, while a small increase would occur under the B1 scenario. At the global scale, the effect of direct CO_2_ fertilization was 91.4 mgC m^−2^/CO_2_ (ppm) under the A2 scenario, implying that about 91.4 mgC m^−2^ would be fixed by plants as NPP by using 1 ppm of atmospheric CO_2_ by the 2090s. The effect of CO_2_ fertilization, however, would start to decline after the 2080 s, with a net reduction in stimulative effect by 8%.

Across different latitudes, the effect of CO_2_ fertilization is highest in low latitudes (127.87 mgC m^−2^/CO_2_ (ppm)), and lowest in high latitude (15.52 mgC m^−2^/CO_2_ (ppm)). Although a higher effect of CO_2_ feritlization is found under the A2 scenario in low latitude regions, this effect would decline after the 2070s with a net reduction in strength by 12.63 mgC m^−2^ during the 2090s compared to the 2070s. Interestingly, the strength of CO_2_ fertilization would increase continuously under the B1 scenario in low latitude regions through the 21^st^ century. The strength of CO_2_ fertilization is lower in mid- and high-latitude regions compared to low latitude regions where NPP shows a continuous increase in response to CO_2_ fertilization through the 21^st^ century.

## Discussion

### 4.1 Comparison of DLEM-simulated NPP with previous estimates

The DLEM-simulated global terrestrial NPP is 54.57 PgC yr^−1^ for the period 2000–2009, which is comparable to MODIS-based estimate of 53.5 PgC yr^−1^ during the 2000s [16], and also falls in the range of 44–66 PgC yr^−1^ as estimated by 17 global terrestrial biosphere models [29]. The large variation in global NPP estimates among these models are primarily caused by different model representations of nutrient and water constraints on NPP in various terrestrial ecosystems. For instance, both field- and model-based studies indicated that carbon and nitrogen are closely interacted in many terrestrial ecosystems [60]. Some of these models do not simulate carbon-nitrogen interaction, which generally result in an overestimate of global NPP. In addition, moisture in root zone is critically important for plants especially in areas under frequent water stress [65], [66]. Remote sensing algorithms, uses a nonlinear function of increasing temperature to estimate production responses to water limitation, which is not capable of accurately accounting for environmental stresses such as rooting depths. Such function can enhance heat stress greatly in low latitude regions, introducing large uncertainties in NPP estimates.

Our results indicate that high latitude biomes have the greatest potential to increase NPP in response to future climate and CO_2_ changes. Mainly due to climate change effect, the boreal needleleaf deciduous forest and tundra would have the largest NPP increase by 79.5% and 78.4% in the 21^st^ century, respectively (Table 2). Hill and Henry [67] found that aboveground biomass of tundra sedge community increased on average by 158% in 2005 compared to the 1980s primarily due to climate warming. Our climate dataset indicates boreal forests and tundra ecosystems would experience 3–6°C temperature increase in the 21^st^ century (Figure 4). In contrast, the model predicted a decline in NPP in tropical forests in the climate-only experiment under the A2 scenario that predicted rapid temperature increase in the 21^st^ century. This prediction is supported by Clark et al. [68]’s long-term study in tropical rain forest, which suggests that temperature increase in tropical regions would suppress forests NPP.

### 4.2 Climate change effects on global NPP in the 21^st^ century

Our climate-only scenario simulations indicate that temperature rise in the 21^st^ century would first promote and then reduce global terrestrial NPP. We found that under the A2 scenario the turning point of global NPP would occur when the global mean temperature reaches about 16.5°C. As temperature crosses 16.5°C, the terrestrial biosphere would show a net reduction in NPP compared to the contemporary period (Figure 8; left panel). Our results also show a levelling-off of terrestrial NPP when the global mean temperature reaches about 15°C under both scenarios. Compared with the contemporary global temperature of ∼13.5°C, our finding justifies the goal of maintaining global warming rate under 2°C by the Copenhagen Accord [69]. However, the temperature value of 15°C should not be treated as a threshold to judge climate effects on ecosystem NPP at regional scale, due to the complex responses of ecosystems to climate change in different regions [70]. Our analysis shows that NPP would increase rapidly in high latitude region (e.g., by 34% under the A2 scenario) while decrease in low latitude region in response to climate warming (Figure 9A). The decline in terrestrial NPP in low latitudes is possibly the result of enhanced autotrophic respiration and increased moisture stress caused by rising temperature [27], [29], [30]. Warming may cause increasing moisture stress resulting in a decline in net nitrogen mineralization, therefore lead to reduction in NPP [25]. In contrast, temperature increase in mid- and high-latitudes can stimulate NPP by alleviating low temperature constraints to plant growth [10], lengthening growing season [13], [71], and enhancing nitrogen mineralization and availability [72].

More extreme precipitation regimes are expected to have a substantial effect on terrestrial NPP during the 21^st^ century, and therefore ecological implications of greater intra-annual variability and extremes in rainfall events have received much attention from the scientific community [73], [74]. Our study suggests that precipitation deficit would result in reduction in terrestrial NPP by 375 gC m^−2^ across eastern United States, South America, Africa, Europe and Southeast Asia under the A2 scenario. The B1 scenario, however, shows a reduction of up to 175 gC m^−2^ with no net change in most of the areas across the globe. Such reduction in terrestrial NPP due to decrease in precipitation has been reported worldwide [16], [75], [76]. Although precipitation is projected to increase during the 21^st^ century under the A2 and B1 scenarios, their spatio-temporal variability is a major factor that determines the magnitude of terrestrial NPP. For instance, we observed a reduction in global precipitation by 8% in 2019 that led to a decline in terrestrial NPP of about 375 gC m^−2^ dominated largely in the tropics. Mohamed et al. [77] found larger NPP decline in tropical regions indicating greater impacts of drought and high sensitivity and weaker physiological adjustment to climate variability particularly precipitation. Such variability in precipitation will induce more decline of NPP in the tropics. In addition, frequent extreme drought may counteract the effects of anticipated warming and growing season extension and reduce terrestrial production [76] in mid- and high-latitude reigons. Increase in drought events is primiarly the result of less frequent but more intense precipitation events which would likely decrease NPP in wetter ecosystems but increase NPP in drier ecosystems [73]. Therefore, a deeper understanding of the importance of more extreme precipitation patterns relative to other global change drivers such as increasing atmospheric CO_2_ concentration, elevated temperature and atmospheric pollution is critical to project future NPP in a changing global environment.

### 4.3 CO_2_ fertilization effect and its interaction with climate change

While climate change may have negative (−4.6% under the A2 scenario) to small positive (2.2% under the B1 scenario) effect on global terrestrial NPP, our Climate plus CO_2_ simulations project notable increase (12% under the B1 scenario to 13.9% under the A2 scenario) in NPP by the end of 21^st^ century. Majority of laboratory studies of tree growth under high CO_2_ concentration show enhanced growth rates, on average, with a 60% increase in productivity as a result of doubling of atmospheric CO_2_ [18]. This explains the prominent CO_2_ fertilization effect found in low latitude, especially under the A2 scenario (Figure 12; Table 1 and 2), which would more than compensate the negative effect (−76.1–−104 gC m^−2^ yr^−1^) of climate change and result in an increase in NPP (132.2–233.6 gC m^−2^ yr^−1^) in tropical forest during the 21^st^ century. Elevated CO_2_ was also found to reduce stomatal conductance and increase water use efficiency in water-limited environments [78]. Our study indicates that CO_2_ fertilization effect might help the Mediteranian evergreen shrubland to overcome severe NPP loss (−15.1%) in response to the projected future drought (Figure 4) due to the poleward shift of subtropical dry zones [79], and resulted increase in shrubland NPP (6%) by the end of the 21^st^ century under the A2 scenario (Table 2).

However, field studies also found large uncertainties related to terrestrial ecosystems’ responses to CO_2_ enrichment. Plant response to elevated atmospheric CO_2_ can be modified by increasing temperature [80], soil nutrient deficiency [81], and tropospheric ozone pollution [82]. Temperature rise in the 21^st^ century could enhance mineralization rate of soil nutrients, which would support higher ecosystem productivity. In low latitudes, however, nutrient leaching rate will also increase due to intensive and increasing precipitation. Our analyses indicate that at the beginning of 21^st^ century, relative effect of CO_2_ fertilization on terrestrial NPP would increase fast; then as a result of CO_2_ saturation and gradually intensified water and nutrient limitations, the effect of CO_2_ fertilization would reach its maximum potential in the 2070s, level off and even decline afterwards (Figure 12).

While climate change would result in large increase in terrestrial NPP in mid- and high-latitude regions due to greater growing season extension compared to low-latitude region [83], the effect of increasing CO_2_ concentration on plant growth will be stronger in low-latitude region during the 21^st^ century. This is primarily because of much stronger photosynthetic response under elevated CO_2_ at high temperatures [84]. The DLEM uses a Farquhar model to examine the response of terrestrial primary production to climate change and increasing atmospheric CO_2_ concentration. The Farquhar model may cause a much stronger CO_2_ enhancement at high temperature compared to low temperatures, resulting in higher NPP response to increasing CO_2_ concentration in low latitude compared to mid- and high-latitude regions. Hickler et al. [85] evaluated the Farquhar photosynthesis model and found that the optimum temperature for primary production shifts to higher temperatures under elevated CO_2_. The DLEM simulates a stronger NPP response to elevated CO_2_ under drier environments because elevated CO_2_ reduces the negative effect of drought on plant growth resulting in higher NPP response in low-latitude region. However, mid- and high-latitude regions are primarily thought to be nitrogen limited. Increase in temperature and precipitation in mid- and high-latitude regions would possibly enhance decomposition of soil organic matter resulting in more nitrogen available for plant uptake. The resultant increase in plant nitrogen uptake with further benefits from elevated CO_2_ would result in an increase in NPP in mid- and high-latitude ecosystems such as eastern Europe, eastern Russia, and parts of China (Figure 9B, 9D). Our regional variation in the response of terrestrial NPP to rising CO_2_ concentration are similar to Kirschbaum [86] who used a modified Farquhar model to simulate the response of photosynthesis to elevated CO_2_.

### 4.4 Uncertainty and future research needs

Several limitations and uncertainties are inherent in this study regarding input data, model parameterization and simulations. Our goal is to investigate impacts of future climate change and elevated CO_2_ concentration on the global terrestrial NPP. We applied one GCMs (CCSM3) climate output together with atmospheric CO_2_ data under two emission scenarios (A2 and B1). Discrepancies existing in different global climate models would lead to different estimation of NPP response as projected climate variables change [87]. Further analysis is helpful to explore the uncertainty ranges by adopting climate projections derived from multiple climate models [29]. In addition, terrestrial NPP response to increasing CO_2_ concentration in the model is based on calibration and parameterization at several Free-Air Concentration Enrichment (FACE) sites based on Ainsworth and Long [88]. Because current long-running FACE experiments are all located in the temperate zone [89], we have very little empirical information available on the response of NPP to elevated CO_2_ in the tropics and high-latitude ecosystems. While parameters were well calibrated based on existing field observations, some processes such as responses of C assimilation/allocation and stomatal conductance to elevated temperature and CO_2_ may change due to plant acclimation [90] and dynamic responses of phenology and growing season length [91], which have not been included in the current DLEM simulations. In addition, we only attempted to quantify the terrestrial ecosystem response to seasonal and interannual climatic variability, and increasing atmospheric CO_2_ concentration, but did not consider how climate induced functional change in ecosystem processes [92] may affect terrestrial NPP. We should be aware of how other environmental factors (e.g. nitrogen deposition, tropospheric ozone, and LCLU change and land management, fertilization and irrigation of croplands) may work together with climate change and elevated CO_2_ to influence terrestrial NPP. Also, disturbance such as timber harvest and cropland abandonment may have a substantial effect on C dynamics at the regional scale [93]. Therefore, future research must take into account additional environmental and human factors such as nitrogen deposition, ozone pollution and land use/land cover change. Furthermore, model representations of ecosystem processes associated with human activities must be improved.

### Conclusion and Implications for Climate Change Policy

The DLEM simulations estimate a mean global terrestrial NPP of 54.57 Pg C yr^−1^ in the first decade of the 21^st^ century, resulting from multiple environmental factors including climate, atmospheric CO_2_ concentration, nitrogen deposition, tropospheric ozone, and LCLU change. Climate change in the 21^st^ century would either reduce global NPP by 4.6% under the A2 scenario or slightly enhance NPP (2.2%) under the B1 scenario. In response to climate change, global NPP would first increase and then decline after global warming exceeds 1.5°C, which justifies the goal of keeping the global warming rate under 2°C by the Copenhagen Accord (2009). These results also support the Accord Acknowledgement, which states that staying below 2°C may not be sufficient and a review in 2015 will be conducted to assess the need to potentially aim for staying below 1.5°C. Terrestrial NPP at high latitude (60°N–90°N) in the Northern Hemisphere would benefit from climate change, but decline at low latitude (30°S–30°N) and the central USA. The CO_2_ fertilization effect would improve global productivity as simulated by the DLEM forced by climate plus CO_2_, showing notable increase in NPP by the end of 21^st^ century (12% under the B1 scenario to 13.9% under the A2 scenario). Ecosystem responses to increasing CO_2_, however, are complex and involve large uncertainties, especially in the tropics. The relative CO_2_ fertilization effect, i.e. change in NPP on per CO_2_ (ppm) bases, was projected to increase quickly, level off in the 2070s, and then even decline by the end of the 2080s, possibly due to CO_2_ saturation and nutrient limitation. Compared to the low emission scenario (B1), earth ecosystems would have a less predictable and thus unfavorable future under the high emission scenario (A2) due to large uncertainties related to the global NPP, which are characterized by stronger spatial variation, more complex temporal dynamics, larger differences in biomes’ responses to global changes, and more dramatic and counteracting impacts from climate and elevated CO_2_.

Our model projections indicate that high emission scenario (A2) will not necessarily negatively affect global terrestrial NPP in the future. However, the A2 scenario describes a future with high ecological uncertainty, the rest of 21^st^ century may experience frequent climate extreme events and increasing ecological risks, and a global ecosystem whose behavior and trend are difficult to predict and therefore can hardly be protected with efficiency. The world under the A2 scenario would likely face more serious challenges in food security and water scarcity given a continually growing world human population. To avoid this scenario, we should shift to the low emission scenario.
